# Inhibition of DNA methyltransferase leads to increased genomic 5‐hydroxymethylcytosine levels in hematopoietic cells

**DOI:** 10.1002/2211-5463.12392

**Published:** 2018-02-23

**Authors:** Borbála Vető, Pál Szabó, Caroline Bacquet, Anna Apró, Edit Hathy, Judit Kiss, János M. Réthelyi, Flóra Szeri, Dávid Szüts, Tamás Arányi

**Affiliations:** ^1^ Institute of Enzymology, RCNS, HAS Budapest Hungary; ^2^ Doctoral School of Molecular Medicine Semmelweis University Budapest Hungary; ^3^ MS Metabolomics Laboratory Core Facility RCNS HAS Budapest Hungary; ^4^ MTA‐SE NAP‐B Molecular Psychiatry and *in vitro* Disease Modeling Research Group Budapest Hungary; ^5^ Department of Psychiatry and Psychotherapy Semmelweis University Budapest Hungary; ^6^ CNRS UMR 6214 INSERM U1083 University of Angers Angers France; ^7^ Present address: Sidney Kimmel Medical College Thomas Jefferson University Philadelphia PA USA

**Keywords:** 5‐aza‐2′‐deoxycytidine, 5‐hydroxymethylcytosine, 5‐methylcytosine, ascorbate, LC‐MS/MS

## Abstract

5‐Hydroxymethylcytosine (5hmC) is produced from 5‐methylcytosine (5mC) by Ten‐eleven translocation (TET) dioxygenases. The epigenetic modification 5hmC has crucial roles in both cellular development and differentiation. The 5hmC level is particularly high in the brain. While 5mC is generally associated with gene silencing/reduced expression, 5hmC is a more permissive epigenetic mark. To understand its physiological function, an easy and accurate quantification method is required. Here, we have developed a novel LC‐MS/MS‐based approach to quantify both genomic 5mC and 5hmC contents. The method is based on the liberation of nucleobases by formic acid. Applying this method, we characterized the levels of DNA methylation and hydroxymethylation in mouse brain and liver, primary hepatocytes, and various cell lines. Using this approach, we confirm that the treatment of different cell lines with the DNA methyltransferase inhibitor 5‐aza‐2′‐deoxycytidine leads to a decrease in 5mC content. This decrease was accompanied by an increase in 5hmC levels in cell lines of hematopoietic origin. Finally, we showed that ascorbate elevates the levels of 5hmC and augments the effect of 5‐aza‐2′‐deoxycytidine without significantly influencing 5mC levels.

**Abbreviations**
5azadC: 5‐aza‐2′‐deoxycytidine
5caC: 5‐carboxylcytosine
5fC: 5‐formylcytosine
5hmC: 5‐hydroxymethylcytosine
5mC: 5‐methylcytosine
DNMT: DNA methyltransferase
IDH: isocitrate dehydrogenase
LC‐MS/MS: liquid chromatography coupled with mass spectrometry
TET: Ten‐eleven translocation

In mammals, CpG dinucleotides are prone to become methylated. CpG methylation plays important roles in transcriptional gene silencing, genomic imprinting, and X chromosome inactivation 1. Methylation patterns are established by the *de novo* methyltransferases DNMT3A and DNMT3B. Existing methylation patterns are preserved during cell divisions by the maintenance DNA methyltransferase DNMT1. This enzyme preferentially binds hemimethylated DNA (methylated only on one strand) and consequently replicates the parental DNA methylation to the newly synthesized DNA strand. DNA demethylation can be executed by demethylase enzymes 2. DNA methylation is considered to be a reversible modification as methylation patterns can be established *de novo*, maintained through generations, or undergo dynamic changes genomewide or at specific loci, reflecting environmental conditions 3, 4, 5, 6.

Recently, the presence of another modified base, 5‐hydroxymethylcytosine (5hmC), was discovered in the central nervous system 7 and in other mammalian tissues 8. 5hmC is a key metabolite in the demethylation process. 5mC levels are fairly constant in different tissues due to CpG methylation (4–5% of all cytosines). In contrast, 5hmC levels are variable with the highest level detected in brain (approximately 1% or more) and 10–100 times lower in other cell types 7, 8 or even lower in iPSCs (0.12%) 4 or cell lines 9.

The DNA demethylase Ten‐eleven translocation (TET) enzymes are Fe^2+^‐ and α‐ketoglutarate‐dependent oxidases, which efficiently convert 5mC into 5hmC both *in vitro* and *in vivo* 2. Furthermore, TET can further oxidize 5hmC to 5‐formylcytosine (5fC) and 5‐carboxylcytosine (5caC) 10. Accordingly, the availability of α‐ketoglutarate, a key molecule of the citric acid cycle, can have significant consequences on the epigenetic control of genome activity and thereby has substantial impact on health and disease. Indeed, recent studies have shown that the catalytic activity of TET proteins is impaired due to mutations in the isocitrate dehydrogenase (IDH) genes leading to the formation of 2‐hydroxyglutarate instead of α‐ketoglutarate in various tumors (reviewed in 11). Ascorbate was also reported to play a role in oxidative demethylation of DNA. It is a substantial factor necessary for the full catalytic activity of TET dioxygenases (DNA demethylase) 12, 13 and also of Jmjc histone demethylases 14; thus, it appears to be a mediator at the interface between the genome and environment 15, 16 The role of ascorbate is the reduction of Fe^3+^ to Fe^2+^, which is necessary for the catalytic activity of these enzymes.

Several methods have been developed to detect covalent DNA modifications (reviewed in 17). Bisulfite conversion was set up for mapping 5mC nucleotides, and it is accepted as the gold standard 18. However, using this chemical conversion it is impossible to distinguish 5mC from 5hmC 19. Affinity enrichment methods are based on the immunoprecipitation of fragmented genomic DNA with 5mC and 5hmC antibodies (MeDIP and hMeDIP) followed by PCR or sequencing, and they are widely used for this purpose. Another method to distinguish the two main covalent DNA modifications is the specific glucosylation of 5hmC combined with MspI digestion, where the glucosylated DNA sites are resistant to cleavage, thus allowing single‐nucleotide 5hmC mapping 20. However, these methods are not sufficiently exact for quantitative analysis 21.

An alternative approach for exact quantification of DNA methylation is the highly reliable and simple global genomic quantification by liquid chromatography coupled with mass spectrometry (LC‐MS/MS). This is considered as the most accurate for simultaneously measuring global 5mC and 5hmC levels in genomic DNA 4, 8 after digesting the genomic DNA into nucleotides by a multienzyme cocktail. We have recently developed an alternative approach based on the liberation of nucleobases by formic acid to quantify genomic 5mC content. Using this method, we have previously reported the highly dynamic nature of DNA methylation 22. Our aim here was to improve this technique by developing genomic 5hmC detection. Furthermore, we intended to investigate the role of 5‐aza‐2′‐deoxycytidine on 5hmC levels in a panel of human cell lines. The FDA‐approved epigenetic drug, 5‐aza‐2′‐deoxycytidine (5azadC or decitabine), is a potent methyltransferase inhibitor 23 that reduces the level of 5mC in genomic DNA. Here, we show that 5‐aza‐2′‐deoxycytidine treatment increases 5hmC levels in hematopoietic cells. Moreover, cotreatment with decitabine and ascorbate allows a decitabine‐dependent increase in 5hmC levels in HeLa cells.

## Materials and methods

### Cell culture

A375 melanoma, A2058 melanoma, HepG2 hepatocarcinoma, HeLa cervix carcinoma, MES‐SA uterine sarcoma, H1650 bronchoalveolar carcinoma, HTR8 placenta, BeWo choriocarcinoma, HL60 promyeloblast, and K562 lymphoblast cell lines were obtained from ATCC. They were cultured either in Dulbecco's modified Eagle's medium (A375, A2058, HeLa, MES‐SA, and H1650 cells), advanced MEM (HepG2 cells), or RPMI‐1640 (HL60, K562, and HTR8 cells) supplemented with 10% fetal bovine serum (FBS), 1% glutamine, 1% penicillin/streptomycin, or F‐12 medium supplemented with 20% FBS (BeWo cells). DT40 cells were cultured in RPMI‐1640 medium (Gibco) supplemented with 7% FBS, 3% chicken serum, 50 μm β‐mercaptoethanol, and penicillin/streptomycin. No ascorbate was added to the culture media. Human iPSCs were differentiated into NPCs as described in Ref. 24. Briefly, iPSC‐derived free‐floating embryoid bodies were treated with human recombinant Noggin and DKK1, SB431542, and cyclopamine for 21 days. After rosette formation, NPCs were dissociated and propagated using FGF2. This protocol gives rise to dorsal telencephalic neural progenitors that can be further differentiated into PROX1 expressing dentate gyrus granule cells.

### Drug treatments

Cells were treated with vehicle (DMSO) or 1 μg·mL^−1^ 5‐aza‐2′‐deoxycytidine for 48 h. When indicated, cells were treated with 100 μg·mL^−1^ ascorbate (approx. 500 μm) for 48 h. Medium was changed after 24 h, and fresh medium was also supplemented with the drugs used for the treatments. DNA was extracted according to the Puregene (158745; Qiagen, Hilden, Germany) protocol.

### Animals and animal studies

C57BL/6J mice were derived from mice purchased from The Jackson Laboratories. Mice were kept under routine laboratory conditions in an approved animal facility. The RCNS, Hungarian Academy of Sciences Institutional Animal Care and Use Committees approved the animal studies. Mouse liver and forebrain were taken out freshly from the animals and frozen. Genomic DNA was prepared with DNeasy Blood & Tissue Kit (Qiagen).

### Primary mouse hepatocyte culture

A total of 12‐ to 16‐week‐old male C57BL/6 mice were used. Primary mouse hepatocyte culture was prepared as previously, except for the addition of the upper layer of matrigel 25. Briefly, mice were anaesthetized (20 mg·kg^−1^ tiletamine, 20 mg·kg^−1^ zolazepam, 12.5 mg·kg^−1^ xylazine, and 3 mg·kg^−1^ butorphanol), and livers were perfused with 75 mL oxygenized perfusion buffer (120 mmol·L^−1^ NaCl, 5.4 mmol·L^−1^ KCl, 0.9 mmol·L^−1^ NaH_2_PO_4_, 26 mmol·L^−1^ NaHCO_3_, 5.6 mmol·L^−1^ glucose, pH 7.4) supplemented with EGTA (0.5 mmol·L^−1^), followed by a second perfusion with 75 mL perfusion buffer without EGTA. Collagenase digestion was performed by 75 mL perfusion buffer supplemented with 2.5 mmol·L^−1^ CaCl_2_ and 0.2 mg·mL^−1^ collagenase (C5138, Sigma). The digested liver was taken out of the abdominal cavity and minced using tweezers, thereby releasing the hepatocytes. Cells were washed in ice‐cold sterile suspension buffer (10 mmol·L^−1^ HEPES, 142 mmol·L^−1^ NaCl, 7 mmol·L^−1^ KCl, pH 7.4), filtered through a 100‐μm mesh membrane and centrifuged for 4 min at 4 °C at 80 ***g***. After one additional washing step, dead cells were removed by Percoll (Sigma) centrifugation. After checking their viability by trypan blue exclusion staining, cells were seeded on 6‐well plates precoated with 5 μg·cm^−2^ collagen I (BD, 356234) at a density of 0.5x10^6^ cells/well in Williams E medium (Gibco) supplemented with hepatocyte thawing/plating supplement (Gibco) and 10% FBS. Cells that did not attach were removed by refreshing the culture medium after 1 h. 24 h postseeding, the medium was renewed.

### MS measurement

DNA was hydrolyzed to nucleobases by chemical means using formic acid as described previously 26. Briefly, 100 μL formic acid (100%) was added to 5–20 μL of samples and pipetted into a 2‐mL glass vial. The tightly crimped vial was kept at 130 °C for 90 min. After nitrogen evaporation, the samples were reconstituted in 80 μL of acetonitrile:water:formic acid 49.5 : 49.5 : 1 solution and pipetted into a 200‐μL microvial. Chemical standards such as dCTP, 5mdCTP, and 5hmdCTP were used to optimize the mass spectrometer to get the highest sensitivity. A Sciex 6500 QTrap mass spectrometer equipped with turboV ion source was used. Perkin Elmer Series200 system (consisting of binary pump, autosampler, and column oven) was used for separation. Water containing formic acid in 0.1% (eluent A) and acetonitrile containing formic acid in 0.1% (eluent B) was used for separation using gradient elution: A/B 30/70(1)‐4.5–90/10(1.5)‐1.5‐30/70(4.5). An Agilent RX‐Sil column (250 × 4.6 mm, 5um) was used for the separation. The flow rate was 1 mL·min^−1^. 40 μL of the samples was injected. The column temperature was ambient, and samples were kept at 5 °C in the autosampler. Source conditions in mass spectrometric measurements were as follows: spray voltage 5000 V, evaporation temperature: 500 °C, curtain, evaporation, and drying gases 45, 45, and 50 instrument units, respectively. 50 ms dwell time and 5 ms pause times were used for each MRM transitions. Collision energy was set to 30 eV.

### Calibration

Calibration was performed on nucleotides. The same sample preparation protocol was applied as for the genomic DNA. A ten‐point calibration curve in the range of 1–10% of mC and a nine‐point calibration curve in the range of 0.01–1% of hmC relative to C were made by mixing the individual nucleotide base solutions. Due to the high dynamic range of the mass spectrometer, the relative concentration values were independent of the absolute amount of nucleotides mixed. While the area ratios of 5mC/C and 5hmC/C were measured, the lowest calibration points were not equal to the lowest limit of quantitation (LOQ). The LOQ and limit of detection (LOD) values depend on the absolute amount of 5hmC. This allowed us to use extrapolated ratios based on relatively strong peaks for integration even if the area ratios were out of the range.

## Results and Discussion

**Figure 1:**
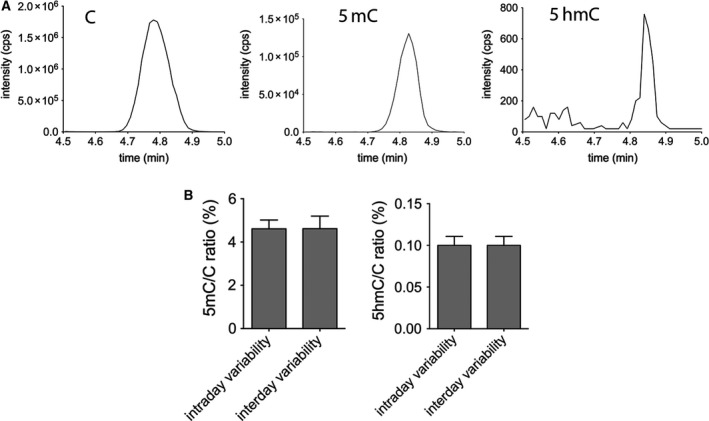
Detection of cytosine derivatives (C, 5mC, and 5hmC) by LC‐MS/MS. Peak intensities from genomic DNA (A) Note the difference of scale. Intra‐ and interday variability (B). Genomic 5mC/C and 5hmC/C ratios (%) in mouse liver are detected by MS. Error bars indicate SD *n* = 8.

We have developed a new HPLC‐MS method to detect 5mC and 5hmC levels in the genome with liquid chromatography–mass spectrometry (LC‐MS/MS). The method uses formic acid to hydrolyze DNA to nucleobases instead of enzymatic digestion often described in the literature 4, 8. Although the retention times of the nucleobases are close to each other, the selectivity of MRM scan mode enables the LC‐MS/MS to detect them selectively according to their different weights of molecular and fragment ions (Fig. 1A). We have not investigated in the present study, but with further improvements, formylcytosine and carboxylcytosine levels could be also detected in the genome by the same approach. The technique is sufficiently sensitive to detect the low levels of 5hmC found in the genome. Next, we examined the variability of the MS measurement. To investigate this, genomic DNA extracted from a C57BL/6 mouse liver was divided into 21 identical samples. The samples were measured by MS at three distinct time points to determine intraday and interday variabilities. Each day seven parallels were measured. Fig. 1B shows the intra‐ and interday variabilities of the MS measurement detecting genomic 5mC/C and 5hmC/C ratios in mouse liver. The coefficients of variation (SD/mean) for intraday variability were 0.08 and 0.10, respectively. Very similar interday coefficients of variation were observed: 0.12 and 0.18, respectively. Although small and similar intra‐ and interday variabilities were determined for both 5hmC/C and 5mC/C measurements, all the parallels of an individual experiment on one cell type presented in this study were always processed together from the beginning (treatment of the cells) to the analysis of DNA methylation and hydroxymethylation levels following MS detection.

**Figure 2:**
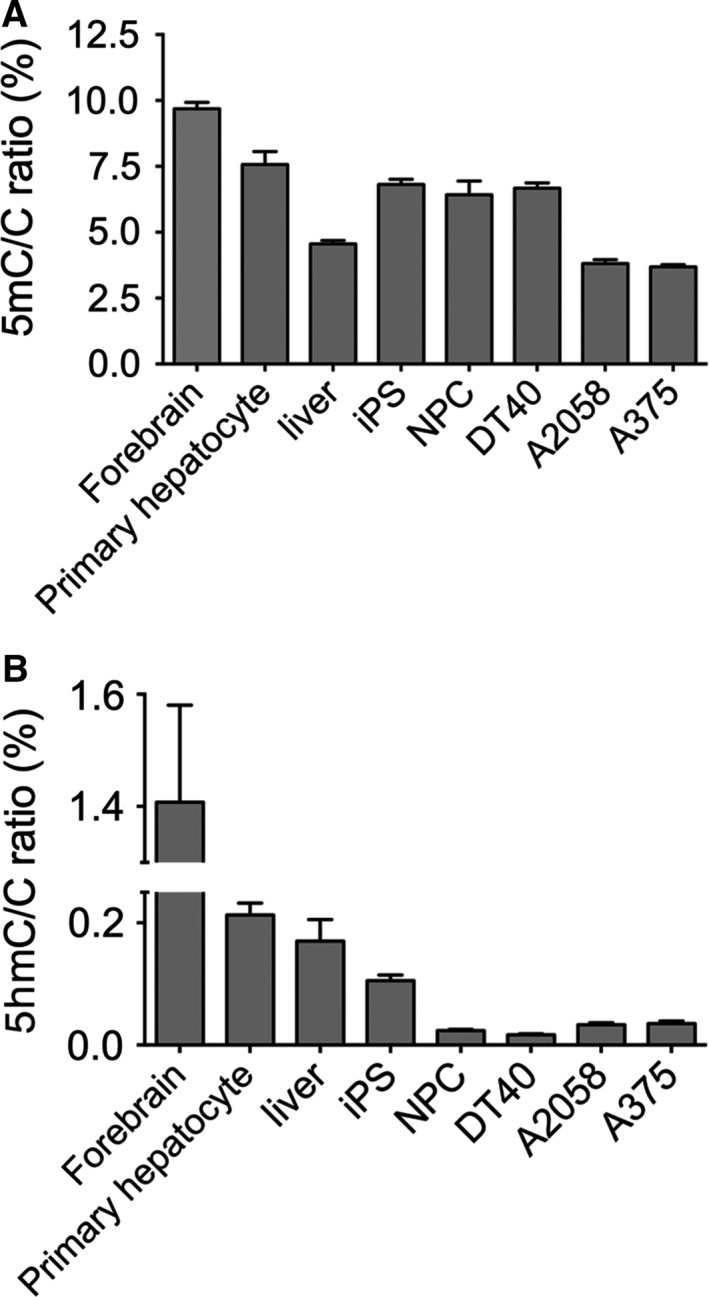
Genomic 5mC/C (A) and 5hmC/C (B) ratios (%) in cell lines, primary cells, and tissues. No ascorbate was added to the culture media. Error bars indicate SD *n* = 4–6. [Corrections added after online publication on 28 February 2018: y‐axis of part (B) amended to 5hmC]

Next, we investigated the genomic 5mC and 5hmC levels in different types of cells and tissues. Fig. 2A shows the detected genomic 5mC/C ratios. It has been shown previously that the tissue‐specific distribution of 5mC is relatively stable among different tissues, but it is highly variable for 5hmC 8. However, we have observed very high 5mC/C ratios in mouse forebrain (9,6%). This high level of DNA methylation probably corresponds to the reported high level of non‐CpG methylation in brain 27, 28. We also observed a slightly higher level of DNA methylation in murine primary hepatocytes relative to the methylation level detected in mouse liver samples, which was similar to the previously reported values 29. The difference might be explained by the loss of nonhepatocyte cells with lower methylation and/or by the hypermethylation of the primary hepatocytes in the culture conditions. This potential *de novo* methylation might also include non‐CpG methylation, as *de novo* methyltransferases efficiently methylate CpA dinucleotides, as well 30, 31. In addition, we observed relatively high (approximately 6,5%) genomic 5mC/C ratios in human‐induced pluripotent stem cells (iPSCs) and their derivatives, neuronal progenitor cells (NPCs). This high level of DNA methylation is typical for iPSCs 32. A similar level of methylation was detected in chicken DT40 B‐cell lymphoma, while the immortalized cancer cell lines (A375 human melanoma, A2058 human melanoma) contained less than 4% 5mC. The highest genomic DNA methylation level was approximately three times higher than the lowest.

A much higher degree of variation of 5hmC levels was detected in our array of cell lines and tissue samples (Fig. 2B). Almost a hundred‐fold more 5hmC was measured in the samples with the highest 5hmC level compared to those with the lowest. In the mouse forebrain, we detected high 5hmC level with a 5hmC/C ratio of 1.4%, which is similar to previous studies on brain‐derived cells 7. In accordance with previous reports, low level of 5hmC was detected in the genome of adult mouse liver 8, 9, 33. Here, we also show similar level of approx. 0.2% 5hmC/C for mouse primary hepatocytes. The iPSCs had a clearly lower 5hmC level than brain and liver cells. Furthermore, a striking difference of 5hmC levels was observed between immortalized cell lines and the samples mentioned above. DT40, A2058, and A375 cells have low genomic 5hmC/C ratios (below 0.06%), confirming that cell culture conditions result in a dramatic reduction in global 5hmC levels 9. As reported previously, differentiating NPCs were also characterized by low 5hmC levels 34, which was similar to that observed in the case of immortalized cells. Altogether, these experiments demonstrate the applicability of our newly developed sensitive and simple method to quantify global 5hmC levels.

**Figure 3:**
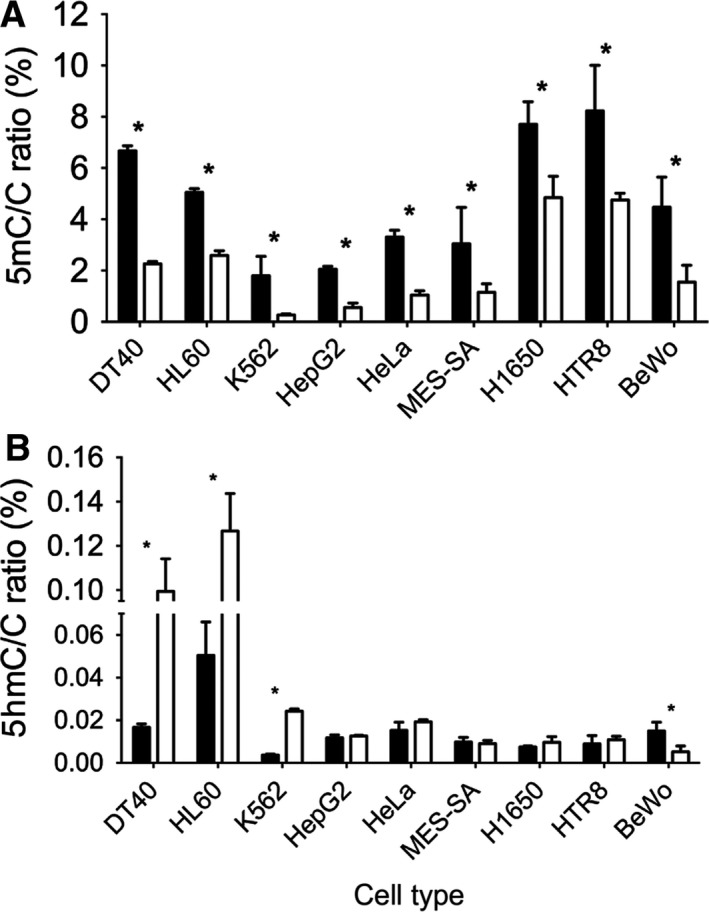
Genomic 5mC/C (A) and 5hmC/C levels (B) detected by LC‐MS upon 5‐aza‐2′‐deoxycytidine treatment. Black and white columns indicate vehicle and 5‐aza‐2′‐deoxycytidine treatment, respectively. No ascorbate was added to the culture media. Error bars indicate SD *P* < 0.05. *n* = 3–5. [Corrections added after online publication on 28 February 2018: y‐axis of part (B) amended to 5hmC]

5azadC is a molecule frequently used to inhibit genomic DNA methylation for research purposes and to treat myelodysplastic syndrome and AML in clinics. A paradoxical increase in the 5hmC level in HL60 human promyeloblast cells upon decitabine treatment has been recently reported 35. The authors explained their observation by the preferential demethylation of hemimethylated relative to methylated DNA strands by the TET enzymes. These strands appear during replication and remain hemimethylated as a result of the inability of the cells to remethylate them due to decitabine treatment. However, the authors did not test any cell types other than HL60. Therefore, to assess whether the described phenomenon is ubiquitous, the effect of the hypomethylating agent on 5hmC level was investigated in a broader range of cell lines in our next set of experiments by taking advantage of the newly developed sensitive technique (Fig. 3). Cells were selected to represent various tissues of origin (hematopoietic, liver carcinoma, uterine carcinoma, melanoma lung carcinoma, and choriocarcinoma). As seen previously, they exhibited variable but mostly low (below 5%) methylation and very low (below 0.05%) 5hmC levels. We have chosen a 48‐h treatment to increase the number of cells undergoing division and the amount of hemimethylated DNA strands in the population investigated. As expected, treatment with the DNA methyltransferase inhibitor 5azadC resulted in systematic and significant decrease in 5mC levels. Upon the 48‐h treatment, 5mC/C ratios diminished to approximately 50% compared to vehicle‐treated cells (Fig. 3A). When we looked at the effect of 5azadC first, we could confirm that the treatment results in a paradoxical increase in 5hmC level in HL60 cells elevating the initial level of 0.05% to 0.12% (Fig. 3B). However, it is necessary to emphasize that the 5mC decrease is much greater than 5hmC increase, as the most important part of 5mC loss is due to the absence of remethylation on the newly synthetized strands and only a small part could be attributed to the TeT activity at hemimethylated loci. Furthermore, 5hmC is less stable and can eventually become further oxidized to other cytosine derivatives, such as carboxylcytosine, formylcytosine, or cytosine 8.

Interestingly, we found that the paradoxical effect of 5azadC on 5hmC levels was only detectable in hematopoietic cells. For instance, in HepG2 cells we could not detect any quantitative 5hmC change. Surprisingly, in other cells of hepatic origin (Huh7) 5azadC treatment was shown to lead to reorganize nuclear 5hmC by immunostaining 36. In the case of BeWo choriocarcinoma, 5azadC treatment resulted in significant loss of genomic 5hmC level. However, we found that the initial observation reported in the literature showing that decitabine treatment leads to increased 5hmC/C ratios was characteristic of hematopoietic cells we investigated. In addition to HL60 human promyeloblast cells, K562 human myelogenous leukemia and DT40 chicken lymphoblastoma cells also showed significantly increased 5hmC levels upon 5azadC treatment (*P* < 0.05, *t‐*test) (Fig. 3). This might suggest that a particular mechanism specific to the hematopoietic cells is responsible for the observed phenomenon. One can hypothesize that expression levels of TeT genes are different in these cells than in the others. It is a conceivable hypothesis that the expression levels of TeT genes are higher in hematopoietic cells *per se*. Alternatively, originally low TeT expression levels could be highly increased upon treatment. However, based on publicly available data (proteinatlas.org 37) none of these seems to be the case. Although K562 cells seem to have high TeT1 expression, they have moderate TeT2 and TeT3 expression levels. HL60 cells have higher TeT2 and TeT3 expression level accompanied with lower TeT1 level. These expression levels were not dramatically different from those of other cells tested in our panel. In particular, BeWo cells had similar expression to K562 with a markedly different effect of 5azadC on their 5hmC levels. Similarly, other related gene expression levels (IDH1 and IDH2 responsible for the synthesis of α‐ketoglutarate or DNMTs) do not allow establishing an obvious correlation between the effect of 5azadC on 5hmC levels in hematopoietic cells and the absence of the effect in cells of different origin.

**Figure 4:**
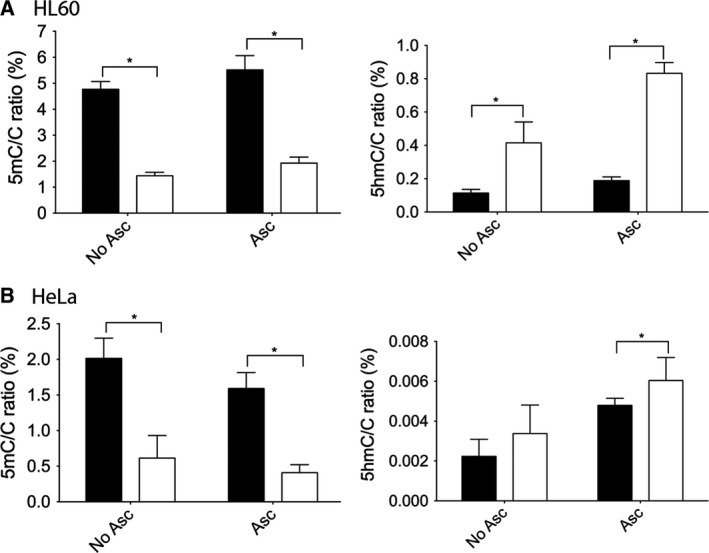
Genomic 5mC/C and 5hmC/C ratios (%) in HL60 (A) and HeLa cells (B). Treatment was performed for 48 h with vehicle or ascorbate (Asc) or ascorbate and 5‐aza‐2′‐deoxycytidine (Aza). Black and white columns indicate vehicle and 5‐aza‐2′‐deoxycytidine treatment, respectively. Error bars indicate SD *P* < 0.05. *n* = 4–5.

We therefore hypothesized that the lack of clear effect of 5azadC on 5hmC level is due to the different oxidative balance in the cells. This might lead to very low activity of TeT enzymes in some cells due to their inability of efficiently reducing Fe^3+^ back to Fe^2+^ at the end of the catalyzed reaction. As stated above, ascorbate was reported to regulate DNA methylation as a substantial cofactor for the full catalytic activity of TET dioxygenases by reducing back Fe^3+^ to Fe^2+^ 12, 13. Therefore, the effect of ascorbate was investigated. Plasma ascorbate concentration was reported to be between 50 and 100 or as high as 200 μmol·L^−1^ 38, 39, 40. As ascorbate has a short half‐life, we used a 100 μg·mL^−1^ (approx. 500 μm) concentration in our experiments. Here, we show that ascorbate treatment of human HL60 and HeLa cells did not induce global DNA methylation changes. Furthermore, cotreatment of the same cells with ascorbate and decitabine did not cause a greater 5mC decrease than decitabine alone. However, the global 5hmC levels became significantly elevated in our experiments on the tested human hematopoietic cells upon ascorbate treatment (Fig. 4A and B). This increase was much more robust than that observed upon 5azadC treatment alone, indicating that the two molecules have synergistic effect, and suggests that even in HL60 cells ascorbate is not present at sufficient level for the full activity of the TeT enzymes. Furthermore, we could demonstrate the increase in 5hmC levels upon 5azadC treatment in HeLa cells only in the presence of ascorbate. Altogether, these data suggest that some cell lines do not have functionally fully active TeT enzymes and are therefore unable to respond to decitabine treatment by 5hmC increase probably due to a scarcity of ascorbate.

Our data reported here demonstrate that decitabine treatment reduces global 5mC and increases 5hmC levels in different cells, particularly those originating from hematopoietic malignancies. There is increasing evidence that DNA hydroxymethylation and TET2 function is highly dysregulated in hematologic malignancies (T‐ALL, AML, CML), suggesting that TET proteins can act as tumor suppressors (reviewed in 41). Indeed, the expression level of TeT2 positively correlated with tumor‐free survival according to a recent study in CLL patients 42. Our data suggest that decitabine treatment in patients with hematopoietic tumors might simultaneously lead to DNA demethylation and increased 5hmC levels, the latter playing a putative positive role in the therapeutic effect of the drug. Recent data suggest that decitabine could also be used efficiently in solid tumors alone or in a combination therapy (for a recent review 43). In these cases, gene‐specific DNA methylation changes were considered due to the treatment, but global effects and potential normalization of 5hmC level might also play a role in the molecular action of the drug. Our results also raise the possibility that cotreatment with dietary ascorbate and decitabine might lead to an increased effect and thereby an increased overall survival in hematopoietic, or potentially in other malignancies.

## Author contributions

TA and DS conceived and designed the project; BV, PS, CB, AA, EH, JK, JMR, and FS acquired the data. TA, DS, PS, and BV analyzed and interpreted the data; TA and BV wrote the manuscript.
